# A Governess Protests!

**Published:** 1888-05-12

**Authors:** 


### The Editor's Letter-Box.

[Our Correspondents are reminded that prolixity is a great bar to publication, and that brevity of style and conciseness of statement greatly facilitate early insertion.]

### A GOVERNESS PROTESTS!

The gentleman who occupied "The Doctor's Pulpit" in last week's issue of The Hospital appears to have a very vague notion of the duties of a governess. He seems to have gone out of his way to throw stones at single women in general, and governesses in particular. Infants of two years of age are, or should be, under the management of a nurse, or mother, in the nursery, certainly not in the school-room. It may be governesses, like George Eliot's parsons, are "too highly learnt for common sense," but perhaps if necessary they might descend to a knowledge of the fact that if a tea-cup were placed within the reach of a child of two, he would probably seize it. A Governess.

